# PReS-FINAL-2029: Long-term follow-up of patients with juvenile idiopathic arthritis (JIA) in a single center: a systematic chart review

**DOI:** 10.1186/1546-0096-11-S2-P42

**Published:** 2013-12-05

**Authors:** BE Bica, DS Bastos, TA Nogueira

**Affiliations:** 1 Rheumatology, Universidade Federal do Rio de Janeiro, Rio de Janeiro, Brazil

## Introduction

Juvenile idiopathic arthritis (JIA) is the most common chronic pediatric rheumatic disease, affecting 4 of every 1,000 children worldwide. It may cause joint damage, persistent pain and retardation of normal growth, which eventually may lead to long-term disability and decreased quality of life. Treatment aims disease control and induction of remission, preventing joint damage and unfavorable outcomes.

## Objectives

To determine the disease outcome of JIA patients followed in a single center in Rio de Janeiro, Brazil.

## Methods

JIA patients registered to the Rheumatology Unit of a University Hospital from 2000 to 2012, first seen before age of 16, and with a follow-up of at least 5 years had their chart data reviewed in the search for information, including: gender, subtype of JIA, age of diagnoses, duration of disease, presence of uveitis, surgery, medication used.

## Results

Ninety-four patients were identified and among these patients, 53 (56.38%) were female and 41 (43.62%) were male with age between nine and forty years. The average duration of disease was 14.7 years, with an average diagnoses gap of one year and five months. Onset subtypes of JIA were: oligoarticular in 29 (30.85%), systemic in 23 (24.47%), entesites-related arthritis in 14 (14.89%), polyarticular RF negative in 14 (15.96%), polyarticular RF positive in 8 (8.51%), psoriatic arthritis in 5 (5.32%). Remission of disease according to Wallace et al (2004) was seen in 73 (77.66%) patients, of which 39 (53.42%) were currently using medication (methotrexate 66,67%, NSAID 43.59%, corticosteroid 41.03%, biological drugs 38.46%, antimalarial drugs 10.26%, leflunomide 7.69%, azathioprine 5.13%, ciclosporin 2,56%). Uveitis occurred in 13 (13.83%) patients. Among the 15 (15.96%) patients who underwent surgery, eight (8.51%) patients had arthroplasty and two (2.13%) had knee tenotomy. Five patients (5.32%) had ophthalmic surgery.

## Conclusion

The study showed remission of disease in 73 (77.66%) patients, diagnosed as oligoarticular onset in 22.31% and systemic in 19.27%. Of those in remission, 39 (39.53%) were currently using some medication. The 21 patients (22.34%) who still presented active arthritis, 7.33% had extended oligoarticular disease and 6.29% had poliarticular disease.

## Disclosure of interest

None declared.

